# Molecular phylogenetics of the *Ophiocordyceps sinensis*-species complex lineage (Ascomycota, Hypocreales), with the discovery of new species and predictions of species distribution

**DOI:** 10.1186/s43008-023-00131-8

**Published:** 2024-02-10

**Authors:** Yongdong Dai, Siqi Chen, Yuanbing Wang, Yao Wang, Zhuliang Yang, Hong Yu

**Affiliations:** 1 Yunnan Herbal Laboratory, College of Ecology and Environmental Sciences, Yunnan University, Kunming, 650504 Yunnan China; 2 School of Basic Medical Science, Guizhou University of Traditional Chinese Medicine, Guiyang, 550025 Guizhou China; 3 CAS Key Laboratory for Plant Diversity and Biogeography of East Asia, Kunming Institute of Botany, Chinese Academy of Sciences, Kunming, 650201 Yunnan China; 4 Yunnan Key Laboratory for Fungal Diversity and Green Development, Kunming Institute of Botany, Chinese Academy of Sciences, Kunming, 650201 Yunnan China; 5 Kunming, China

**Keywords:** Multi-locus phylogeny, *Ophiocordyceps megala*, Species distribution modelling, Biodiversity corridor

## Abstract

*Ophiocordyceps sinensis* is a famous traditional Chinese medicine adapted to the alpine environment of the Qinghai-Tibet Plateau and adjacent regions. Clarification of the species diversity of *Ophiocordyceps sinensis* and its relatives could expand the traditional medicinal resources and provide insights into the speciation and adaptation. The study is prompted by the discovery of a new species, *O. megala*, described here from a biodiversity hotspot in the Hengduan Mountains, China. Combined morphological, ecological, and phylogenetic evidence supports its distinctiveness from *O. sinensis*, *O. xuefengensis*, and *O. macroacicularis*. Additionally, based on the phylogenetic construction of *Ophiocordyceps,* a special clade was focused phylogenetically on the more closely related *O. sinensis* complex, which was defined as the *O. sinensis-* species complex lineage*.* A total of 10 species were currently confirmed in this lineage. We made a comprehensive comparison of the sexual/asexual morphological structures among this species complex, distinguishing their common and distinctive features. Furthermore, using the method of species distribution modelling, we studied the species ocurrences in relation to climatic, edaphic, and altitudinal variables for the eight species in the *O. sinensis*-species complex, and determined that their potential distribution could extend from the southeastern Qinghai-Tibet Plateau to the Xuefeng Mountains without isolating barrier. Thus, the biodiversity corridor hypothesis was proposed around the *O. sinensis*-species complex. Our study highlights the phylogeny, species diversity, and suitable distribution of the *O. sinensis*-species complex lineage, which should have a positive implication for the resource discovery and adaptive evolution of this unique and valuable group.

**Supplementary Information:**

The online version contains supplementary material available at 10.1186/s43008-023-00131-8.

## INTRODUCTION 

*Ophiocordyceps* (Hypocreales, Ophiocordycipitaceae) is a large genus with 324 accepted species names (http://www.speciesfungorum.org/). It was originally introduced for species of *Cordyceps* with asci with conspicuous apical caps and whole (not fragmenting) ascospores with distinct septation (Petch 1924, 1931).

The majority of species in *Ophiocordyceps* possess firm, darkly pigmented stromata or subiculum, especially those with *Hirsutella* asexual morphs while some species produce brightly coloured stromata with *Hymenostilbe* and *Paraisaria* asexual morphs. The stromata are usually tough, wiry, fibrous, or pliant. Perithecia are superficial to completely immersed, oblique, or ordinal in arrangement. Ascospores are usually cylindrical, multiseptate, either disarticulating into part spores or remaining intact after discharge (Sung et al. 2007).

Species of *Ophiocordyceps* are distributed worldwide in forest ecosystems of the tropics and subtropics (Petch 1931; Kobayasi 1941; Tzean et al. 1997; Ban et al. 2015; Luangsa-ard et al. 2018; Wang et al. 2018; Araújo et al. 2014, 2018, 2020; Mongkolsamrit et al. 2019; Zha et al. 2021). Although tropical and subtropical areas have the richest species diversity of *Ophiocordyceps*, alpine or plateau regions cannot be ignored either. *Ophiocordyceps sinensis,* the Chinese caterpillar fungus, is pre-eminant as a rare traditional and valuable Chinese medicine, and is endemic to the Qinghai–Tibetan Plateau (QTP) and its surroundings in high altitude cold environment (Winkler 2008; Li et al. 2011; Zhang et al. 2012; Hu et al. 2013; Xia et al. 2017; Dai et al 2020).

Considering the uniqueness and specificity of *O. sinensis*, the diversity of its relatives may be a valuable key to unlocking new understanding of speciation, adaptation and origin of functional component. What, where, and how to form are undoubtedly of scientific importance. Over the last two decades, we have conducted a large-scale survey of entomopathogenic fungi in the alpine regions of southwestern China, one of the world's biodiversity hotspots (Chen et al. 2013a, b; Dai et al 2020; Wang et al. 2020, 2021; Dong et al. 2022; Sun et al. 2022). Some specimens with huge sclerotia (parasite on a large moth) with long stromata were collected which proved to be a new taxon closely related to *O. sinensis* was identified. We present a morphological description and phylogenetic analysis of this new fungus and assess the species diversity and potential distribution of the *O. sinensis-* species complex lineage.

## MATERIALS AND METHODS 

### Specimen collection

Specimens were collected from Lanping county, Yunnan province, China (26.46° N, 99.17° E, altitude 2500 m), in July 2015 by Hong Yu, Yong-dong Dai, Run-de Yang and Tian-Lin He, parasiting larvae cadavers of *Endoclita* sp. (Hepialidae). All specimens were deposited in the Yunnan Herbal Herbarium (YHH) of Yunnan University, China.

### Fungal isolation and culture

The surface of specimens was rinsed with sterile water, and surface-sterilized with 75% ethanol for 1–3 min. Fresh tissue from internal part of sclerota was transfererd to potato dextroseagar (PDA) and cultured at 20 °C in the dark. After purification, cultures were transferred to PDA slants and stored at 4 °C; isolates were deposited in the Yunnan Fungal Culture Collection (YFCC) of Yunnan University, China.

### Optical and scanning electron microscope

Specimens collected in the field were photographed and measured using a stereomicroscope (Olympus SZ61). Cultures on slants were transferred to PDA plates and cultured in an incubator for three weeks at 20 °C. Ccircular agar blocks ca. 5 mm diam from plates were cut out and placed on new PDA plates for morphological examination.

For the morphological description, microscope slide cultures were prepared by placing a small piece of mycelium on a 5 mm diam PDA block overlain by a cover slip. Micro-morphological observations and measurements were made using an Olympus CX40 microscope.

For scanning electron microscopy (SEM), 1 cm wide agar blocks were cut from PDA cultures, fixed with 4% glutaraldehyde at 4 °C overnight, washed three times with a phosphate buffer solution (PBS; 137 mM NaCl, 2.7 mM KCl, 8.1 mM Na_2_HPO_4_, 1.5 mM KH_2_PO_4_, pH 7.4) three times, for10 min each time. Fixed hyphae and conidia were dehydrated using a 50%, 70%, 90% and 100% alcohol series, with 10 min ateach level; and finally dehydrated with supercritical carbon dioxidet. Tthe samples were placed in SEM stubs,,coated with gold–palladium. Conidia and mucilage were examined with scanning electron microscope (S-3400N, Hitachi, Japan) and photographed.

### DNA extraction, PCR amplification and sequencing

The genomic DNA of the fungus and its host were extracted with a Fungi DNA isolation Kit according to the manufacturer’s instructions (TransGen Biotech, Beijing, China) from the stroma and the surface of sclerotium sections respectively. Genomic DNA was also extracted from the fungal pure cultures (0.05–0.1 g axenic mycelia). The genomic DNA (> 20 ng/μL) was used as the template to amplify DNA fragment.

Six nuclear loci of the fungus were amplified and sequenced, including the internal transcribed spacer (ITS), small and large subunit ribosomal RNAs (nrSSU, nrLSU), transcription elongationfactor-1 alpha (*tef*), and the largest and second largest subunits of RNA polymerase II (*rpb1* and *rpb2*). The mitochondrial cytochrome coxidase subunit I (*cox1*) sequences of the insect hosts were also amplified and sequenced. The polymerase chain reaction (PCR) assay was conducted using the manufacturer’s manual. The primer information used is provided in Additional file 1: Table S1. PCR products were sequenced on the ABI3700 automatic sequence analyzer (Sangong, Shanghai). The sequences were newly added from seven species and their host insects: *Ophiocordyceps megala*, *O. sinensis*, *O. laojunshanensis, O. lanpingensi*, *O. nujiangensis, O. xuefengensis,*and *O. liangshanensis*.

### Molecular phylogeny

To construct a phylogenetic tree for *O. megala* and related species, and recongnize the diversity of *the O. sinensis*-species complex, representative taxa with five loci (nrSSU, nrLSU, *tef*, *rpb1* and *rpb2*) were collected from previously published phylogenetic studies of the genus (Sung et al. 2007; Quandt et al. 2014; Sanjuan et al. 2015; Ban et al. 2015, Simmons et al. 2015; Mongkolsamrit et al. 2019). Five loci of each sample were retrieved from GenBank.

A 5- locus dataset was established combining preiously published data with their new sequences generated for the present study. A total of 185 taxa with 5-locus sequence data were selected to represent the diversity of *Ophiocordyceps* (Table 1). Three *Tolypocladium* species were chosen as the out groups (Kepler et al. 2014). The ITS region was used to compare the phylogenetic difference among the *O. sinensis-* species complex.

**Table 1:** Specimen and their 5 genes accession numbers information of nrSSU, nrLSU, *tef*, *rpb1* and *rpb2*

Species	Speciemen	Accession numbers				
		nrSSU	nrLSU	*tef*	*rpb1*	*rpb2*
***Ophiocordyceps megala***	**YHH OMLP1507001**	**NMDCN00011VK**	**NMDCN00011VM**	**NMDCN00011VO**	**NMDCN00011VQ**	**NMDCN00011VS**
***O. megala***	**YHH OMLP1507002**	**NMDCN00011VL**	**NMDCN00011VN**	**NMDCN00011VP**	**NMDCN00011VR**	**NMDCN00011VT**
*Hirsutella cryptosclerotium*	ARSEF_4517	KM652066	KM652109	KM651992	KM652032	
*Hirsutella fusiformis*	ARSEF_5474	KM652067	KM652110	KM651993	KM652033	
*Hirsutella gigantea*	ARSEF_30		JX566977	JX566980	KM652034	
*Hirsutella guyana*	ARSEF_878	KM652068	KM652111	KM651994	KM652035	
*Hirsutella illustris*	ARSEF_5539	KM652069	KM652112	KM651996	KM652037	
*Hirsutella kirchneri*	ARSEF_5551	KM652070	KM652113	KM651997		
*Hirsutella lecaniicola*	ARSEF_8888	KM652071	KM652114	KM651998	KM652038	
*Hirsutella liboensis*	ARSEF_9603	KM652072	KM652115			
*Hirsutella necatrix*	ARSEF_5549	KM652073	KM652116	KM651999	KM652039	
*Hirsutella nodulosa*	ARSEF_5473	KM652074	KM652117	KM652000	KM652040	
*Hirsutella radiata*	ARSEF_1369	KM652076	KM652119	KM652002	KM652042	
*Hirsutella rhossiliensis*	ARSEF_3207	KM652079	KM652122	KM652005	KM652044	
*Hirsutella rhossiliensis*	ARSEF_2931	KM652078	KM652121	KM652004	KM652043	
*Hirsutella rhossiliensis*	ARSEF_3751	KM652081	KM652124	KM652007	KM652046	
*Hirsutella rhossiliensis*	ARSEF_3747	KM652080	KM652123	KM652006	KM652045	
*Hirsutella satumaensis*	ARSEF_996	KM652082	KM652125	KM652008	KM652047	
*Hirsutella* sp1	OSC_128575	EF469126	EF469079	EF469064	EF469093	EF469110
*Hirsutella* sp2	ARSEF_2348	KM652077	KM652120	KM652003		
*Hirsutella strigosa*	ARSEF_2197	KM652085	KM652129	KM652012	KM652050	
*Hirsutella subulata*	ARSEF_2227	KM652086	KM652130	KM652013	KM652051	
*Hirsutella thompsonii*	ARSEF_256	KM652090	KM652135	KM652018	KM652053	
*Hirsutella versicolor*	ARSEF_1037	KM652102	KM652150	KM652029	KM652063	
*O. acicularis*	OSC_110987	EF468950	EF468805	EF468744	EF468852	
*O. acicularis*	OSC_110988	EF468951	EF468804	EF468745	EF468853	
*O. agriota*	ARSEF_5692	DQ522540	DQ518754	DQ522322	DQ522368	DQ522418
*O. amazonica*	Ophama2026	KJ917562	KJ917571	KM411989	KP212902	KM411982
*O. aphodii*	ARSEF_5498	DQ522541	DQ518755	DQ522323		DQ522419
*O. appendiculata*	NBRC_106959	JN941729	JN941412	AB968578	JN992463	AB968540
*O. appendiculata*	NBRC_106960	JN941728	JN941413	AB968577	JN992463	AB968539
*O. araracuarensis*	HUA 186148	KC610790	KF658679	KC610739	KF658667	KC610717
*O. arborescens*	NBRC_105890	AB968387	AB968415	AB968573		AB968535
*O. arborescens*	NBRC_105891	AB968386	AB968414	AB968572		AB968534
*O. blattarioides*	HUA186093	KJ917559	KJ917570	KM411992	KP212910	
*O. brunneipunctata*	OSC_128576	DQ522542	DQ518756	DQ522324	DQ522369	DQ522420
*O. buquetii*	HMAS_199617	KJ878940	KJ878905	KJ878985	KJ879020	
*O. cf. acicularis*	OSC_128580	DQ522543	DQ518757	DQ522326	DQ522371	DQ522423
*O. clavata*	NBRC_106961	JN941727	JN941414	AB968586	JN992461	AB968547
*O. coccidiicola*	NBRC_100682	AB968391	AB968419	AB968583		AB968545
*O. cochlidiicola*	HMAS_199612	KJ878917	KJ878884	KJ878965	KJ878998	
*O. coenomyia*	NBRC 108993	AB968384	AB968412	AB968570		AB968532
*O. crinalis*	GDGM_17327	KF226253	KF226254	KF226256	KF226255	
*O. curculionum*	OSC_151910	KJ878918	KJ878885		KJ878999	
*O. elongata*	OSC_110989		EF468808	EF468748	EF468856	
*O. entomorrhiza*	KEW_53484	EF468954	EF468809	EF468749	EF468857	EF468911
*O. evansii*	Ophsp.858	KC610796	KC610770	KC610736	KP212916	
*O. formicarum*	TNS_F18565	KJ878921	KJ878888	KJ878968	KJ879002	KJ878946
*O. formosana*	MFLU:15–3888	KU854951		KU854949	KU854947	
*O. formosana*	NTU_00035			KT275192	KT275190	KT275191
*O. forquignonii*	OSC_151902	KJ878912	KJ878876		KJ878991	KJ878945
*O. fulgoromorphila*	HUA 186139	KC610794	KC610760	KC610729	KF658676	KC610719
*O. fulgoromorphila*	Ophara729	KC610795	KC610761	KC610730	KF658677	AB968554
*O. geometridicola*	BCC35947	AB104725	KJ878877		KJ878992	
*O. geometridicola*	BCC79823				MH028163	MH028173
*O. gracilioides*	Ophuni866	KC610799		KC610742	KF658674	KC610718
*O. gracilioides*	Ophgrc934	KJ917556		KM411994	KP212914	
*O. gracilis*	EFCC_3101	EF468955	EF468810	EF468750	EF468858	EF468913
*O. gracilis*	EFCC_8572	EF468956	EF468811	EF468751	EF468859	EF468912
*O. gracillima*	Ophgrc679		KC610768	KC610744	KF658666	
*O. heteropoda*	NBRC 100642	JN941720	JN941721	AB968594		AB968555
*O. highlandensis*	HKAS83207	KM581284			KM581274	KM581278
*O. hignland*	YHH_OH1301	KR479869		KR479870	KR479872	KR479874
*O. irangiensis*	OSC_128578	DQ522556	DQ518770	DQ522345	DQ522391	DQ522445
*O. karstii*	MFLU:15–3884	KU854952		KU854945	KU854943	
*O. karstii*	MFLU:15–3885	KU854953		KU854946	KU854944	
*O. konnoana*	EFCC_7315	EF468959		EF468753	EF468861	EF468916
*O. lanpingensis*	YHOS0707	KC417459	KC417461	KC417463	KC417465	
*O. lloydii*	OSC_151913	KJ878924	KJ878891	KJ878970	KJ879004	KJ878948
*O. longissima*	TNS_F18448	KJ878925	KJ878892	KJ878971	KJ879005	
*O. longissima*	NBRC 106965	AB968392	AB968420	AB968584		AB968546
*O. macroacicularis*	NBRC_100685	AB968388	AB968416	AB968574		AB968536
*O. macroacicularis*	NBRC_105889	AB968390	AB968418	AB968576		AB968538
*O. macroacicularis*	NBRC_105888	AB968389	AB968417	AB968575		AB968537
*O. melolonthae*	OSC_110993	DQ522548	DQ518762	DQ522331	DQ522376	
*O. myrmecophila*	CEM1710	KJ878928	KJ878894	KJ878974	KJ879008	
*O. myrmecophila*	TNS_27120	KJ878929	KJ878895	KJ878975	KJ879009	
*O. neovolkiana*	OSC_151903	KJ878930	KJ878896	KJ878976		
*O. nigrella*	EFCC_9247	EF468963	EF468818	EF468758	EF468866	EF468920
*O. nooreniae*	BRIP 55363	KX673811	KX673810	KX673812		KX673809
*O. nutans*	OSC_110994	DQ522549	DQ518763	DQ522333	DQ522378	
*O. nutans*	NBRC_100944	JN941713	JN941428	AB968588		AB968549
*O. nujiangensis*	YFCC 8880	ON723384	ON723381	ON868820	ON868823	ON868826
*O. nujiangensis*	YHH 20041	ON723385	ON723383	ON868822	ON868825	ON868827
*O. pauciovoperitheciata*	BCC45562		MH028162			MH028181
*O. pauciovoperitheciata*	BCC39781		MH028167	MH028182		
*O. ponerinarum*	HUA186140	KC610789	KC610767	KC610740	KF658668	
*O. pseudoacicularis*	BCC49256		MH028154		MH028166	MH028176
*O. pseudoacicularis*	BCC53843		MH028156		MH028168	MH028177
*O. pulvinata*	TNS-F 30044	GU904208	AB721305	GU904209	GU904210	
*O. purpureostromata*	TNS_F18430	KJ878931	KJ878897	KJ878977	KJ879011	
*O. ramosissimum*	GZUH2012HN2	KJ028013		KJ028016	KJ028018	
*O. ramosissimum*	GZUHHN8	KJ028012		KJ028014	KJ028017	
*O. ravenelii*	OSC_151914	KJ878932		KJ878978	KJ879012	KJ878950
*O. rubiginosiperitheciata*	NBRC_106966	JN941704	JN941437	AB968582	JN992438	AB968544
*O. rubiginosiperitheciata*	NBRC_100946	JN941705	JN941436	AB968581	JN992439	AB968543
*O. sinensis*	YN09-64	JX968028	JX968033	JX968018	JX968008	JX968013
*O. sinensis*	YN07-8	JX968027	JX968032	JX968017	JX968007	JX968012
*O. sinensis*	XZ06-44	JX968026	JX968031	JX968016	JX968006	JX968011
*O. sinensis*	QH06-197	JX968025	JX968030	JX968015	JX968005	JX968010
*O. sinensis*	QH09-201	JX968024	JX968029	JX968014	JX968004	JX968009
*O. sobolifera*	NBRC 106967	AB968395	AB968422	AB968590		AB968551
*O. sobolifera*	KEW_78842	EF468972	EF468828		EF468875	EF468925
*O. spataforae*	NHJ_12525	EF469125	EF469078	EF469063	EF469092	EF469111
*O. sphecocephala*	NBRC 101416	JN941698	JN941443		JN992432	
*O. stylophora*	NBRC_100948	JN941693	JN941448	AB968580	JN992427	AB968542
*O. stylophora*	NBRC_100949	JN941692	JN941449		JN992426	
*O. stylophora*	OSC_111000	DQ522552	DQ518766	DQ522337	DQ522382	DQ522433
*O. stylophora*	OSC_110999	EF468982	EF468837	EF468777	EF468882	EF468931
*O. stylophora*	NBRC_100947	JN941694	JN941447	AB968579	JN992428	AB968541
*O. tettigonia*	GZUHCS14062709	KT345955		KT375440	KT375441	
*O. tiputini*	Ophsp 1465	KC610792	KC610773	KC610745	KF658671	
*O. tricentri*	NBRC 106968	AB968393	AB968423	AB968593		AB968554
*O. unilateralis*	OSC_128574	DQ522554	DQ518768	DQ522339	DQ522385	DQ522436
*O. unitubercula*	YHH HU1301	KY923213		KY923215	KY923217	KY923219
*O. unitubercula*	YFCC HU1302	KY923214		KY923216	KY923218	KY923220
*O. variabilis*	ARSEF_5365	DQ522555	DQ518769	DQ522340	DQ522386	DQ522437
*O. xuefengensis*	GZUH2012HN14	KC631789		KC631793	KC631798	
*O. xuefengensis*	GZUH2012HN13	KC631787		KC631792	KC631797	
*O. yakusimensis*	HMAS_199604	KJ878938	KJ878902		KJ879018	KJ878953
*Ophiocordyceps* sp1	TNS_16252	KJ878941	KJ878906	KJ878986		
*Ophiocordyceps* sp2	NHJ_12581	EF468973	EF468831	EF468775		EF468930
*Ophiocordyceps* sp3	TNS_16250	KJ878942		KJ878987	KJ879021	
*Ophiocordyceps* sp4	OSC_110997	EF468976		EF468774	EF468879	EF468929
*Ophiocordyceps* sp5	NHJ_12582	EF468975	EF468830	EF468771		EF468926
*Ophiocordyceps* sp6	TNS_F18550	KJ878911	KJ878875	KJ878959		
*Podonectria citrina*	TNS_F18537		KJ878903	KJ878983		KJ878954
*Torrubiella pruinosa*	NHJ_12994	EU369106	EU369041	EU369024	EU369063	EU369084

The sequences of *Ophiocordyceps megala* were submitted to NMDC (National Microbiology Data Center), and the NMDC accession numbers were list. The others sequencs accession numbers were obtained from GenBank

Sequence alignment of the nrSSU, and nrLSU regions was individually conducted using MAFFT (Katoh et al. 2002). The triplet codon style was set when aligned the exon regions of *tef*, *rpb1* and *rpb2*,*.* ensuring that the sequence can be translate to a protein sequence. The alignments were checked visually and adjusted manually where required. Alignment lengths were 4515 bp, 1109 for nrSSU, 1026 for nrLSU, 931 for *tef*, 553 for *rpb1*, and 935 for *rpb2*. All five loci were combined into a single dataset and 11 data partitions were defined: one each for nrSSU and nrLSU plus nine for each of the three codon positions for the protein coding genes *tef*, *rpb1* and *rpb2*.

The best partitioning scheme and evolutionary models for 11 pre-defined partitions were selected using PartitionFinder2 (Lanfear et al. 2017), with greedy algorithm and the AIC criterion. The following five partitions were identified:Partition. 1—nrSSU, nrLSU, Partition 2—*tef* codon1, *tef* codon2. Partition 3— *rpb1* codon1, *rpb2* codon 1. Partition 4— *rpb1* codon2, *rpb2* codon2. and Partition 5—*tef* codon3, *rpb1* codon3, *rpb2* codon3. A Maximum Likelihood (ML) phylogenetic tree was inferred using IQ-TREE (Nguyen et al. 2015) for 2000 ultrafast (Minh et al. 2013) bootstraps, as well as the Shimodaira–Hasegawa–like approximate likelihood-ratio test (Guindon et al. 2010). The entire phylogenetic construction process was conducted in PhyloSuite (Zhang et al. 2020).

A Bayesian Inference phylogenetic tree was inferred using MrBayes 3.2.6 (Ronquist et al. 2012) under partition model (2 parallel runs, 50,000,000 generations), in which the initial 25% of sampled data were discarded as burn-in. The operation stop rule was set when the average standard deviation of split frequencies was below 0.01. The convergence of the runs was checked using Tracer v.1.6 (Rambaut et al. 2014). Due to the huge amount of data and the time-consuming process, we used the online platform (CIPRES, https://www.phylo.org/portal2/) to complete the calculations. The contree file was visualized with FigTree v.1.6 (http://tree.bio.ed.ac.uk/software/figtree/).

In addition, the ITS sequences were used to clarify the phylogenetic relationships among the *O. sinensis-* complex. The *cox1* sequences were used to identify their host insects. The whole process of these two datasets were conducted in PhyloSuite (Zhang et al. 2020).

### Species distribution modelling

Species occurrence data were mainly collected from our ongoing field studies. Bioclim variables were downloaded from the CliMond Archive (https://www.climond.org/) (Kriticos et al. 2012).

A total of 32 species occurrence data were collected (Table 2). And a total of 35 typical climate, edaphic and altitudinal variables at a grid resolution of 10' were obtained from the CRU CL2.0 dataset. These factors contain the core set of 19 variables (temperature and precipitation), and an extended set of 16 solar radiation and soil moisture variables at a global extent (Additional file 1: Table S2). All climate variables data sources were from the period 1961—1990 (30 years centred on 1975).

**Table 2:** The species occurrence data of eight species within *O. sinensis* -species complex

Species	Distribution		Species	Distribution	
	Longitude	Latitude		Longitude	Latitude
*Ophiocordyceps laojunshanensis*	99.10°	28.93°	*O. karstii*	105.74°	28.36°
	98.95°	28.85°	*O. lanpingensis*	98.97°	26.94°
	99.52°	29.25°		99.14°	26.49°
	99.33°	27.10°		99.20°	26.46°
	98.75°	27.76°		99.26°	26.50°
	100.74°	27.27°		99.16°	26.25°
	100.12°	27.05°		99.21°	26.84°
	100.90°	26.67°		99.02°	26.95°
*O. xuefenensis*	110.41°	27.07°		99.51°	26.64°
	110.68°	27.19°		99.42°	26.76°
	110.53°	27.08°		99.34°	26.47°
*O. liangshanensis*	103.57°	28.26°		99.52°	27.33°
	104.15°	28.41°		99.57°	26.67°
	104.17°	28.46°	*O. macroacicularis*	110.51°	26.76°
*O. megala*	107.03°	28.13°		106.67°	26.39°
	121.19°	24.01°	*O. nujiangensis*	98.87°	27.13°

Species distribution modelling was based on species redundance with MaxEnt V3.4.1 (Phillips et al. 2006; Elith et al. 2011). Randomly, 25% of the data points were extracted as the test data, and “do jackknife to measure variable importance” was selected. The output grid format was set as “cloglog.” The it was visualized with Global mapper17 (https://www.bluemarblegeo.com).

## RESULTS

### Molecular phylogeny

**Fig. 1:**
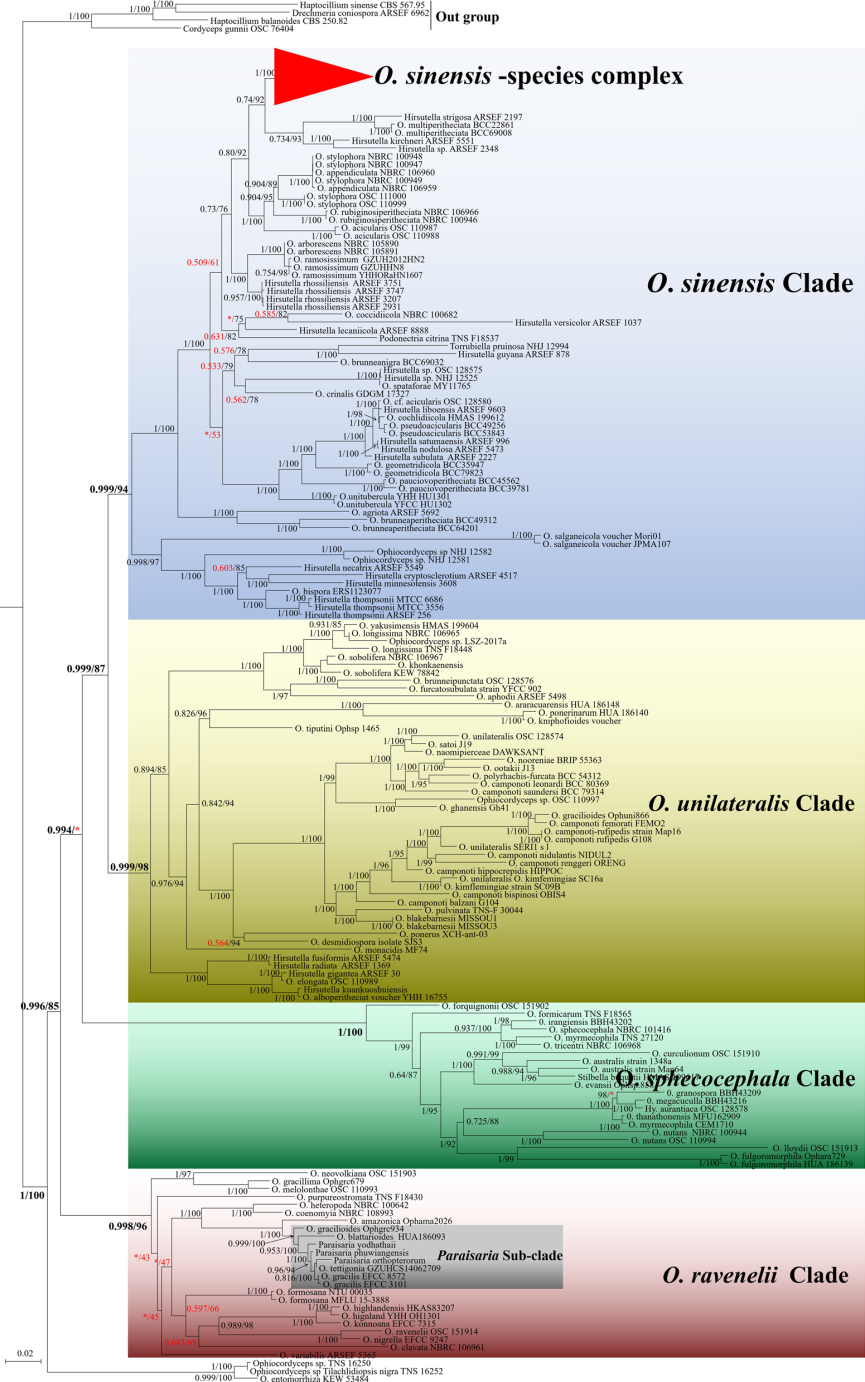
The phylogeny of *Ophiocordyceps* with emphasized on *O. sinensis* -species complex lineage based on 5-locus of nrSSU, nrLSU, *tef*, *rpb1* and *rpb2* datasets. The degree of confidence i lower 0.7 (BPP) and 70 (MLBS) highlight red

Both ML and BI analyses of the combined 5-locus (nr*SSU*, nr*LSU*, *tef*, *rpb1* and *rpb2*) dataset recognized five statistically well-supported clades from a total of 185 taxa within *Ophiocordyceps*, designated here as the *O. sinensis* Clade (MLBS = 94, BPP = 0.999), *O. unilateralis* Clade (MLBS = 87, BPP = 0.999), *O*. *sphecocephala* Clade (MLBS = 100, BPP = 1.00), and *O. ravenelii* Clade (MLBS = 96, BPP = 0.998) (Fig. 1).

In the phylogenetic tree of *Ophiocordyceps* constructed with 5 genes data, a special clade were focused on that had close phylogenetic relationship with *O. sinensis*. We defined it as the *O. sinensis-* species complex lineage (MLBS = 100, BPP = 1.0).

**Fig. 2:**
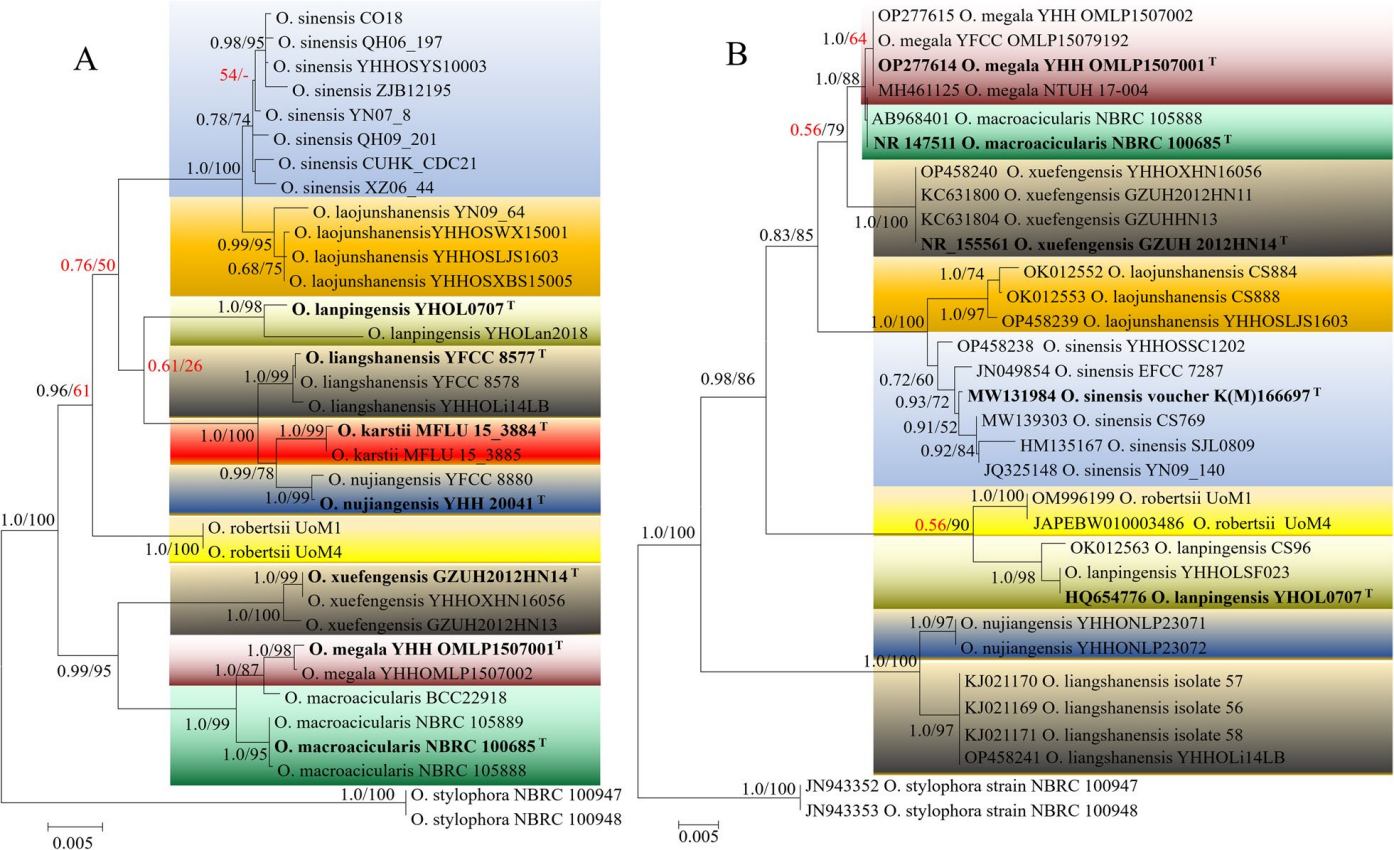
The phylogenetic structure of *O. sinensis*—species complex based on two datasets. **A** 5-locus of nrSSU, nrLSU, *tef*, *rpb1* and *rpb2* datasets; **B** ITS gene. *Notes*: the holotype of each species was marked with superior characters T

After sufficient integration, we confirmed that the clade currently contains 10 species, including *O. megala* reported in this study (Fig. 2). Among them, *O. laojunshanensis* was the nearest to *O. sinensis*. While *O. liangshanensis*, *O. karstii* and *O. nujiangensis* had closed relationships. Our reported *O. megala* was closely related to *O. macroacicularis* and *O.xuefenensis.* By contrast, the location of *O.robertsii* and *O.lanpingensis* were less certain, which phylogentic position was significant difference in 5-locus and ITS datasets.

### Host identification

**Fig. 3:**
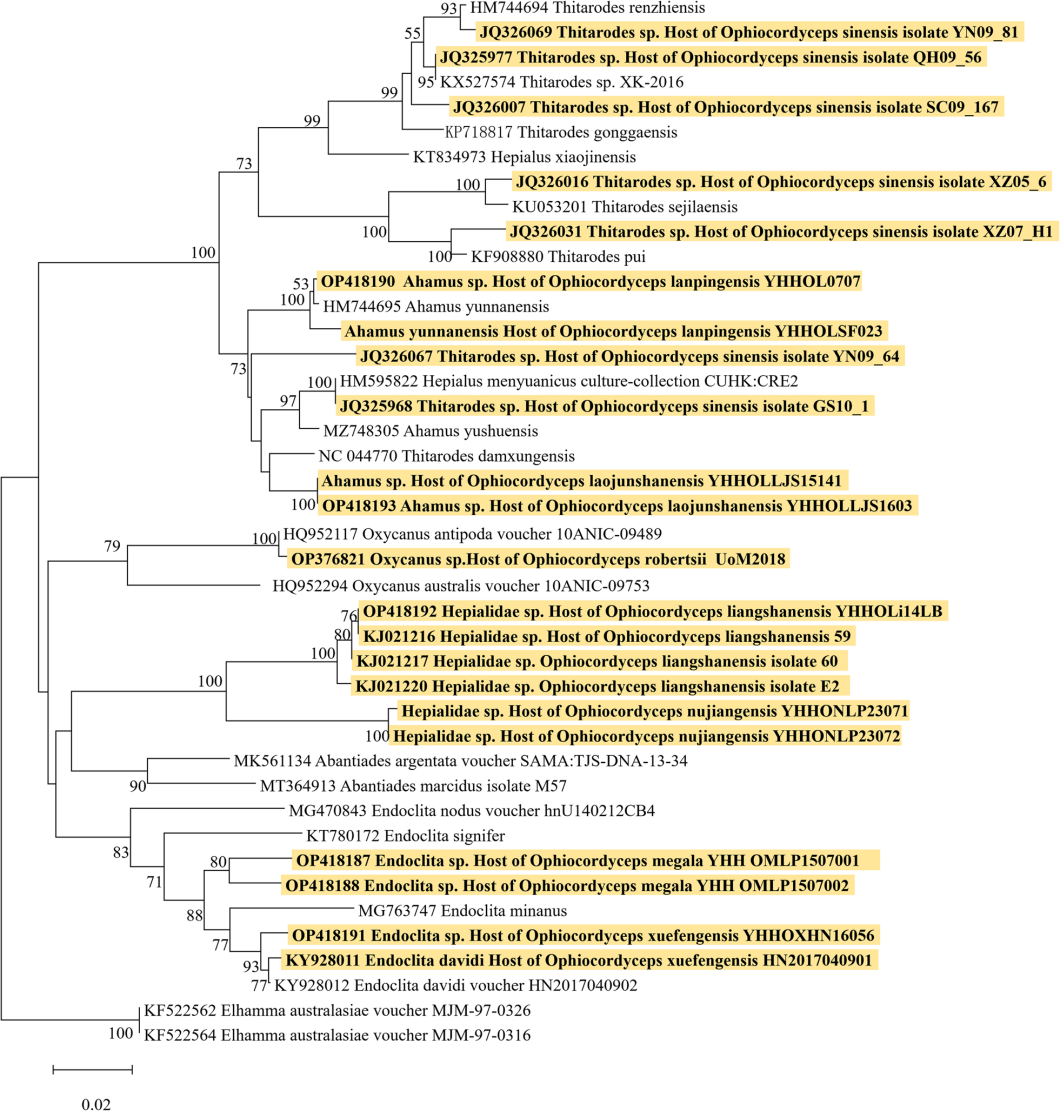
The host identification of of *O. sinensis* -species complex based on *cox1* sequences data

Hosts of *O. megala* and others eight *O. sinensis-* species complex were identified based on the *cox1* gene. The ML tree showed that all hosts belonged to Hepialidae (Lepidoptera), but scattered in different phylogenetic clade (Fig. 3). The host of *O. megala* was identified as *Endoclita* sp. and near to *Endoclita davidi* (the host of *O. xuefengensis*). *Ophiocordyceps sinensis* itself has a very high diversity of hosts (Dai et al 2019), with several clades forming in *Thitarodes* and *Ahamus* (Hepialidae). the host of *O. lanpingensis* and *O. laojunshanensis* were located within these clades.

### Taxonomy

**Fig. 4:**
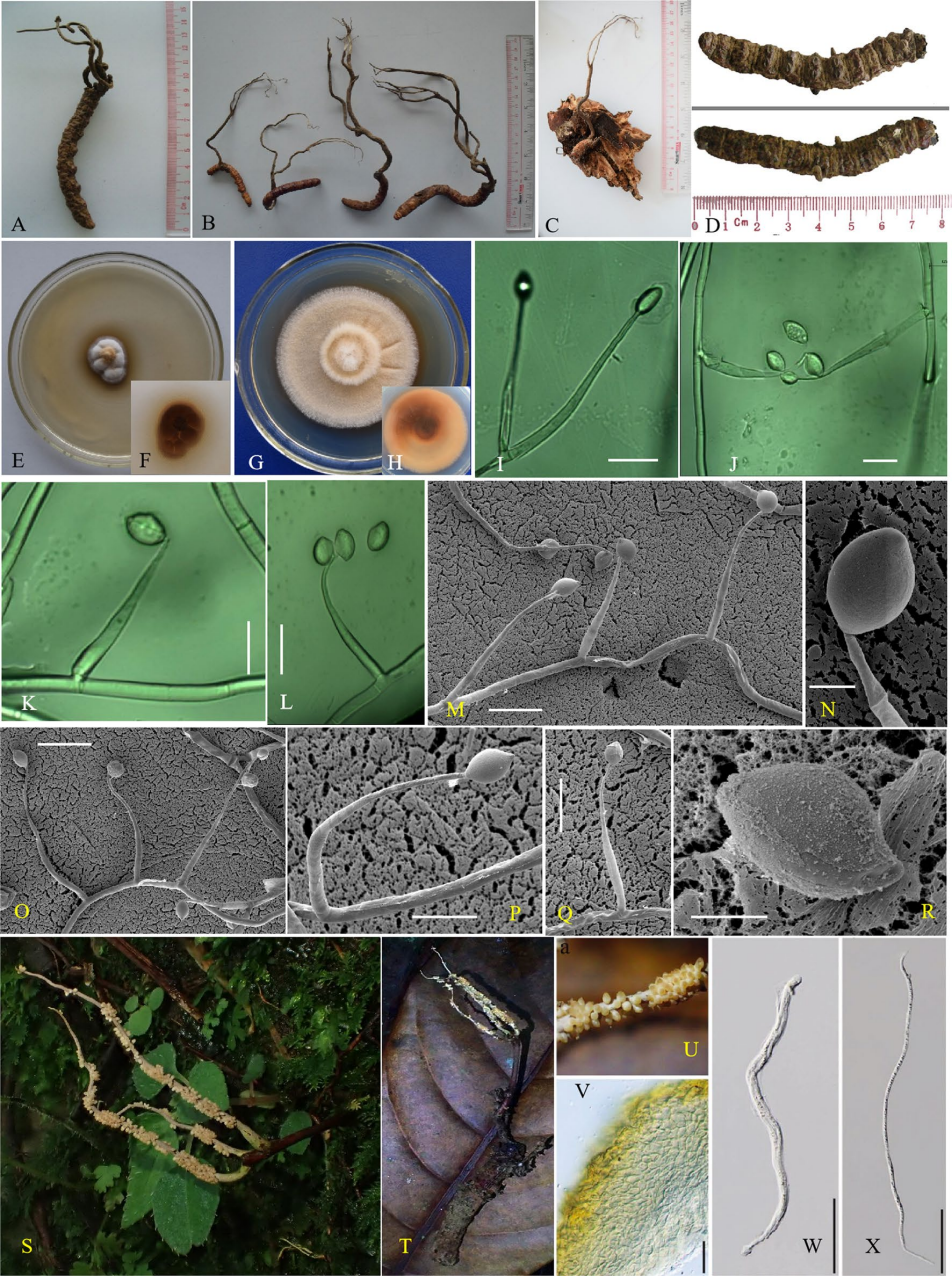
The morphiological and micromorphological characteristics of *Ophiocordyceps megala.* **A**–**C**, **S**–**T**. Wild morph (**A**. holotype YHH OMLP1507001, **C**. Paratype YHH OMSF1601, **S**–**T**. Specimen NTUH 17–004); **D**. Host morph; **E**–**H**. Colony (YFCC OMLP15079192); **I**–**L**. Phialide and conidium; **M**–**R**. Phialide and conidium under electron microscope. Scale bar: 10 μm (**I**–**Q**), 5 μm (**R**), 20 μm (**V**), 50 μm (**W**–**X**)

***Ophiocordyceps megala*** Hong Yu bis & Y.D. Dai **sp. nov.** (Fig. 4).

#### MycoBank

MB845561

#### Etymology

After the long and large (massive) stromata and huge host.

#### Diagnosis

Differs from related species mainly in having massive stromata, long phialides, large single conidia, and the huge-sized host.

#### Type

**China:** *Yunnan Province*: Lanping County, Yingpan village, 26.46° N, 99.17° E, alt. 2800 m, on a larva of *Endoclita* sp. burried in soil, Jul 2015, H. Yu, R.D. Yang, & Y.D. Dai (holotype– YHH OMYP 1507001, ex-holotype cuture– YFCC OMYP 15079192) (Fig. 4A, D). *Yunnan Province*: Shuifu County, Taiping village, 28.40° N, 104.1° E, alt. 2300 m, on a larva of *Endoclita* sp. in the plant root, Aug 2016, *H. Yu, R.D. Yang, & Y.D. Dai* (paratype–YHH OMSF1601) (Fig. 4C).

#### Description

*Asexual morph*: *Hirsutella*-like. *Colonies*– on PDA reaching 18–23 mm diam after 3 wk at 20 °C, round, irregular swell, grey-white to pale brown. Hyphae grow regularly, slowly forming a raised hyphal circle. *Hyphae*–hyaline, septate, branched, smooth-walled, 2.6–4.5 μm wide. *Conidiogenous cells*–arising from hyphae directly or laterally, monophialidic, hyaline, smooth-walled, subulate, tapering gradually into slender neck, 46.9–75.6 µm long, base 3.2–4.5 µm wide and neck 1.0–1.5 µm wide. *Conidia*– arising singly from the apex of the conidiogenous cells, oval or citriform shape, usually single, rare 2(-3) aggregated. 8–12 × 5–7 µm.

#### Stromata

Single, stipe clavate, solid, lignified, yellow–brown, arising from the head of host, 80–320 mm long, several small branches from tips, greyish white.

#### Sexual morph

*Ascomata*– lavate, terminal, no infertile tip. *Perithecium–*superficial, long ovoid, about 180–200 μm. *Asci*–Hyaline, cylindrical, eight-spored ascus, about 190–250 μm, apex thickened to form ascus cap. *Ascospore*– linear, needle-shaped, multi-septate with indistict septation, about 240 μm (Ariyawansa et al. 2018).

#### Host

On larvae of *Endoclita* sp. *(Lepidoptera, Hepailidae).* thick and solid, 19–27 × 80–130 mm.

*Distribution and Habitat***:** China (Yunnan Province and Taiwan Province), also Myanmar. Lived in the subtropical broadleaf forest.

#### Additional specimens examined

**China**– *Yunnan Province*: Lanping County, Yingpan village, 26.46° N, 99.17° E, alt. 2800 m, on a larva of *Endoclita* sp. in soil, Jul 2015, *H. Yu, R.D. Yang & Y.D. Dai* (YHH OMYP 1507002); Shuifu County, Taiping village, 28.40° N, 104.1° E, alt. 2300 m, on a larva of *Endoclita* sp. in the plant root, Aug 2016, *H. Yu, R.D. Yang, & Y.D. Dai* (YHH OMSF1602-05) (Fig. 4B). *Taiwan Province*, Cueifong, Nantou County, 24.13° N, 121.19° E, 9 Jul 2017, Wei-Yu Chuang (NTUH 17–004, Fig. 4S-V) (Ariyawansa et al. 2018). **Myanmar**– *Kachin state*: Muse, alt. 2650 m, Jul 2014, *H. Yu & J.M. Xiao* (YHH OMM1401-05) (Additional file 2: Figure S1).

#### Notes

Specimens with mature sexual structures were not found among the many specimens of *O. megala* used in this study. However, a specimen numbered NTUH 17-004 previously identified as *O. macroacicularis* had the same ITS sequence characteristics with *O. megala* (Fig. 2B). Their 28S ribosomal RNA gene fragment (~ 800 bp) (MH461122) was also total the same (Ariyawansa et al 2018). Meanwhile, NTUH 17-004 had significant difference in the stromata, peritheciua and asci compared with the holotype of *O. macroacicularis*, but it was highly consistent with *O. megala*, indicating that the specimen NTUH 17-004 should be treated as *O. megala*. Thus, the ascus data could be supplied based on this specimen, it provided extremely important circumstantial evidence for the description of the new species *O. megala* (Fig. 4S–X).

**Fig. 5:**
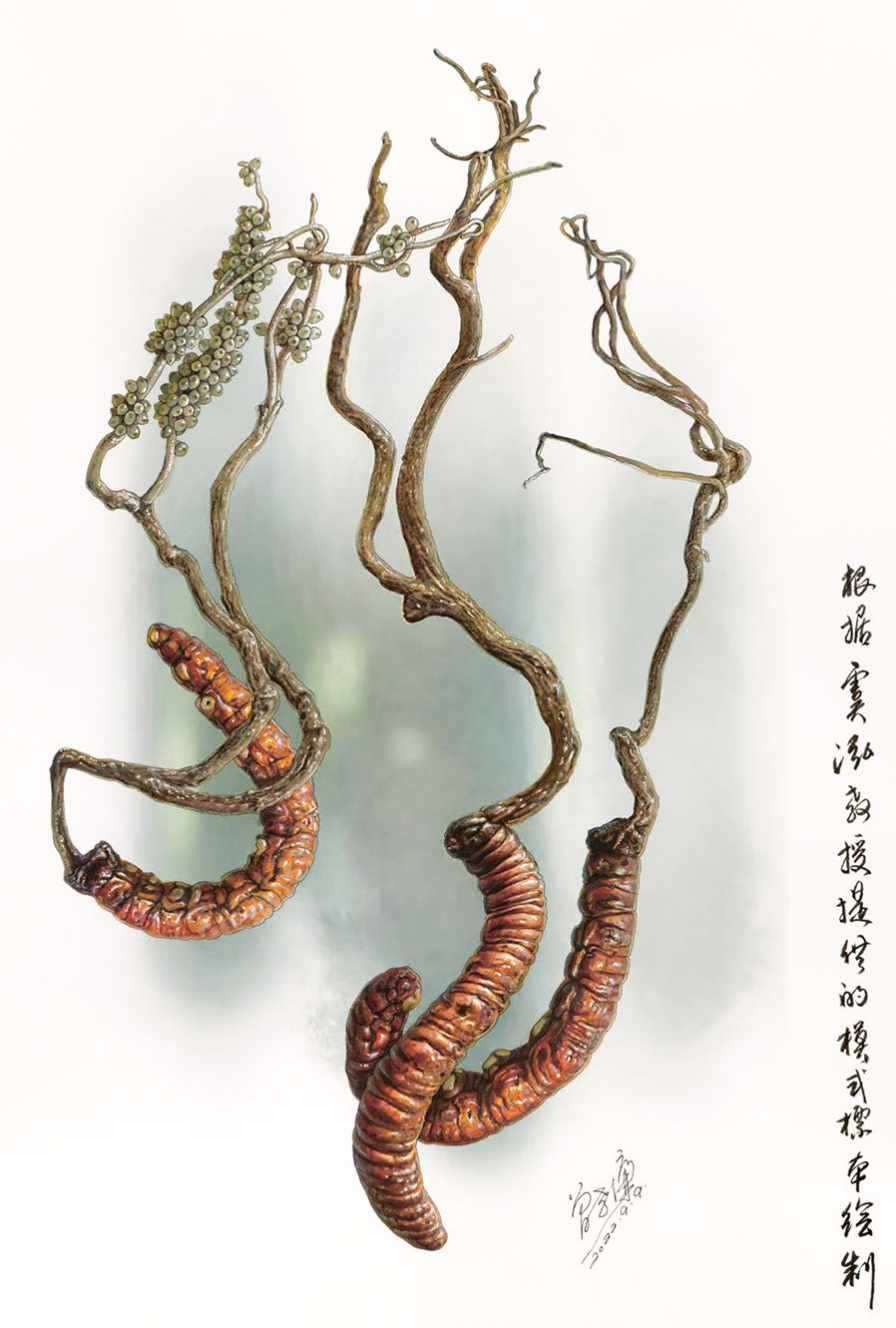
The hypothetical illustration of *Ophiocordyceps megala* with sexual structures (drawing by Zeng Xiaolian)

Based on these characteristics, we illustrated a hypothetical *O. megala* with sexual structures (Fig. 5).

## Species clarification of the *O. sinensis-*species complexs

**Fig. 6:**
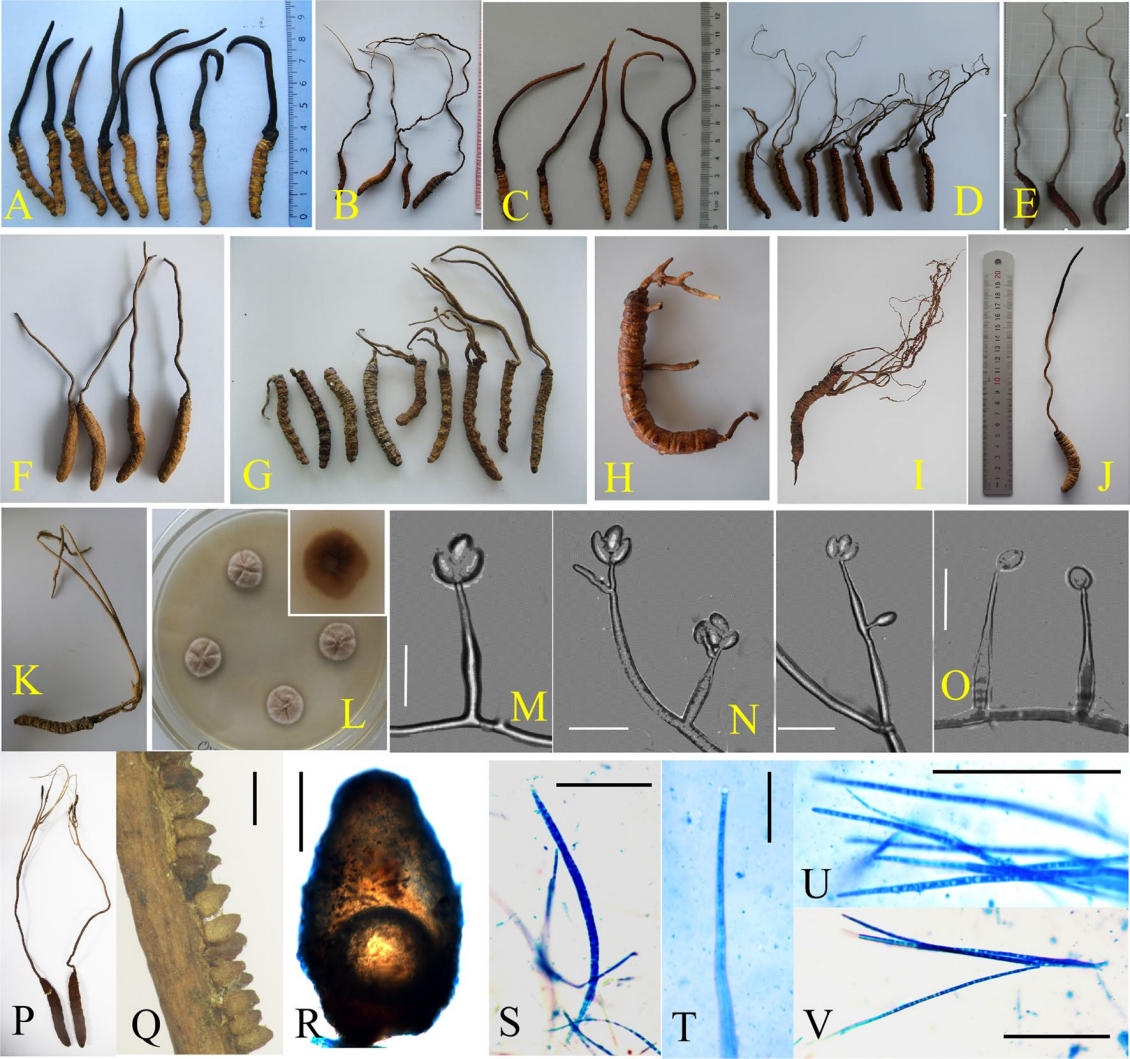
The morphiological characterastics comparision of ten species within *O. sinensis*-species complex. *A.O. sinensis*; B. *O. karstii*; C. *O. laojunshanensis*; D. *O. lanpingensis*; E,P–V. *O. nujiangensis*; F. *O. liangshanensis*; G. *O. megala*; H,K–O. *O. xuefenensis*; I. *O. macroacicularis*; J. *O. robertsii.* L. Colony of *O. xuefenensis*, M–O*.* Phialide and conidium of *O. xuefenensis*; Q. Ascomata of *O. nujiangensis*; R. Perithecium of *O. nujiangensis*; S-V: Asci and Ascospore of *O. nujiangensis.* Scale bar: 2 μm (M–O), 500 μm (Q), 200 μm (R), 100 μm (S-V)

Based on the phylogeny, we clarified 10 species in the *O. sinensis*-species complex. To conduct a more comprehensive sexual/asexual characteristics comparison, the detailed description of the sexual morphs of *O. nujiangensis* and asexual morphs of *O. xuefengensis* were supplied in our present study, as this was lacking in the previous papers (Wen et al. 2013; Sun et al. 2022) (Fig. 6). And on this basis, we could summarize some common characteristics of this lineage.

### Sexual stage

*Stroma–* wooden, linear, mostly yellowish brown to taupe. *Ascomata—*clavate, terminal, with or without infertile tip. *Perithecium—*superficial, ovoid. *Asci—*cylindrical, apex thickened to form ascus cap. *Ascospore—*linear, thread-like, needle-shaped, multi-septate with indistict septation,

### Asexual stage

*Hirsutella*-like. The colony slow growing on PDA and hard in texture. Mostly brown to dark brown. *Conidiogenous cells—*arising from hyphae directly or laterally, monophialidic, hyaline, smooth-walled, subulate, tapering gradually into slender neck. *Conidia—*arising from the apex of the conidiogenous cells, oval or citriform shape, usually single, rare 2(-3) aggregated.

### Host

Hepialidae (Lepidoptera). Ahamus, Endoclita, Thitarodes, Oxycanus, Abantiades.

### Habitat

Alpine forests, aipina meadow, subtropical broad-leaved forest, bamboo forest.

In addition to commonalities, we conducted the list of sexual-asexual morphological comparison among *O. sinensis*-species complex (Table 3), the differences between traits were quantified, which could be used as species clarification and retrieval. There were great differences among hos, perithecia and asci. Significant differences can also be found in Conidiogenous cells and Conidia.

**Table 3:** Morphological comparison of ten species among *Ophiocordyceps sinensis-* species complex

Species	Host	Habitat	Stromata	Perithecium	Asci	Ascospore	Colony	Conidiogenous cells	Conidia	References
*O. megala*	*Endoclita* sp. (Hepialidae)	In tree hole or tree root	Rust, cylindrical, solitary or branched, 80–320 mm long	Superficial, long ovoid, about 180–200 μm	Cylindrical,about 190–250 μm	Needle-shaped, multiseptate with indistict septation, 100–200 μm	18–23 mm diameter on PDA at 20℃ in 3 weeks	Monophialidic, clavate, swollen base and taper neck. base (3.2–4.5 µm wide) and slender neck (1.0–1.5 µm wide) (*Hirsutella*-like), 46.9–75.6 µm long	Oval, 8–12 × 5–7 µm	In this study
*O. sinensis*	Thitarodes and Ahamus (Hepialidae)	Soil in alpine meadow and scrub	Single, occasionally 2–3, 40– 110 mm long	Nearly superficial, ellipsoidal to ovate, 380–550 × 140–240 µm	Slender, long, 240–485 × 12–16 µm	Usually 2–4 mature ascospores, multiseptatewith indistict septation, 160– 470 × 5–6 µm	Grew slowly, 20 mm in diameter after 3 week, on PDA at 15 °C	Hyaline,smooth, tapered neck, 10.47 ~ 28.56 µm	Oval, 7.19 ~ 10.31 × 3.31 ~ 4.87 µm	Liang (2007); Li et al. (2021)
*O. laojunshanensis*	*Ahamus* sp. (Hepialidae)	soil in alpineforest	Single,clavate, slender, 47.0–93.0 mm long	globoid, 200–300 × 200–350 μm. arranged loosely	clavate, 165.0–275.0 × 11.5–14.5 μm	hyaline, filiform, septate, 130.0–250.0 × 5.0–6.0 μm (Fig. 1a–f)	grew very slowly, only up to 6–10 mm in diameter after 2 months on PDA at 16 °C	hyaline, with verrucose, acerate, (*Hirsutella*-like) 15–39(–50) μm long	long elliptic, 6.0–13.5 × 3.0–4.0 μm	Chen et al. (2011)
*O. xuefengensis*	*Endoclita davidi* (Hepialidae)	In tree hole or tree trunk of *Clerodendrum cyrtophyllum*	Yellowish-brown, solitary or several, 140–460 × 2–7 mm	Superficial, long ovoid, 416–625 × 161–318 μm	Cylindrical, 191–392 × 4.5–8.9 μm	Thread-like, needle-shaped, multiseptate with indistinct septation, 130–380 × 1.4–5.2 μm	16 × 20 mm diameter on PDA at 20℃ in 3 week, gray-white colony with ray-like shape	Swollen base (3.0–4.1 µm wide) and slender neck (1.0–1.5 µm wide) (*Hirsutella*-like) (Hirsutella-like), 31–78 µm long	8–12 × 5–7 µm, common 3–5 aggregated	Wen et al. (2013); In this study
*O. liangshanensis*	*Abantiades* (Hepialidae)	Soil in the Qiong bamboo forest	Single, or occasionally branched, cylindrical, solid, 200–300 × 1.5–2.5 mm long	Superficial, long ovoid, 450–740 × 300–450 µm	Hyaline, cylindrical, 260–480 × 8–12 µm	170–240 × 3.1–4.1 µm, with many septa, 5.5–20.0 × 2.5–4.1 µm	12–15 mm diam on PDA after 2 months, hard, round, irregular swell, brown	Phialidic with swollen base (3.8–4.7 µm wide) and slender neck (2.0–3.0 µm wide) (*Hirsutella*-like), generating on hyphae laterally or terminally, hyaline, 46.9–75.6 µm long	Oval, 8.0–12.6 × 3.6–5.0 µm, 1–2(-4) aggregated	Zang et al. (1982); Wang et al. (2021)
*O. macroacicularis*	Hepialidae	Soil near the root of *Fallopia japonica*	Solitary or branched,97.2–166.1 × 1.3–2.4 mm	Oval, light brown, 410–760 × 260–420 μm	Hyaline, cylindrical, 235–310 μm long	Needle-shaped, multiseptate with indistinct septation, 200–300 × 2.3-3 μm. (8-)14–16(-20) septa	20 mm diameter on PSA at 25℃ in 2 weeks	Swollen base (2.9–4.1 µm wide) and slender neck (1.0–1.3. µm wide) (Hirsutella-like), 30.4–42.0 µm long	3.0–5.0 × 5–8.0 µm long	Ban et al. (2015)
*O. lanpingensis*	*Ahamus* sp. (Hepialidae)	Soil	Slender, Several, or solitary, 50–160 × 0.2–1.3 mm	Superficial, ovoid, 310–370 × 200–240 µm	Cylindrical, 240–300 × 5.1–6.5 µm	Filiform, 240–300 × 1.4 µm. septa, 3.3–4.9 × 1.1–1.4 µm	16–18 mm diameter on PDA at 20℃ in 2 weeks	(Hirsutella-like), swollen base and slender neck, 12.5–24.0 × 3.7–5.1 µm	Oval, 2(-4) aggregated, 7.3–9. 6 × 3.3–6.9 µm	Chen et al. (2013a, b, 2011)
*O. nujiangensis*	Hepialidae sp	Soil	Solitary, 148–182 mm long	Superficial, ovoid, 600–750 × 380–4600 µm	Cylindrical, 220–300 × 8–11 μ m	eedle-shaped, multiseptate with indistinct septation, 190–270 × 3–5 μm	20–11 mm diam on PDA after 14 weeks, hard	Swollen base (3.6–4.9 µm wide) and slender neck (1.0–1.5 . µm wide) (Hirsutella-like),54.9–76.5 µm long	Oval or fusiform, 6.4–11.2 × 3.7–6.4 µm long	Sun et al. 2022; In this study
*O. karstii*	Hepialidae sp.	Soil in the bamboo forest	Solitary, 140-145 mm long	600–765 × 247–323 μ m	186–228 × 8–12 μ m	Needle-shaped, multiseptate with indistinct septation, 173–202 × 3–5 μm	Not observed	Not observed	Not observed	Li et al. (2016)
*O. robertsii*	Hepialidae sp	Soil in the broad-leaf forest	Solitary, 180–200 mm long	Perithecia dense, superficial, long ovoid, 600–680 × 500–550 µm	300–450 μm long	210–330 × 3.3–4.4 µm, with many septa	Not observed	Not observed	Not observed	Cunningham (1921); Xu et al. (2023)

## Suitable distribution of the *O. sinensis*-species complex

**Fig. 7:**
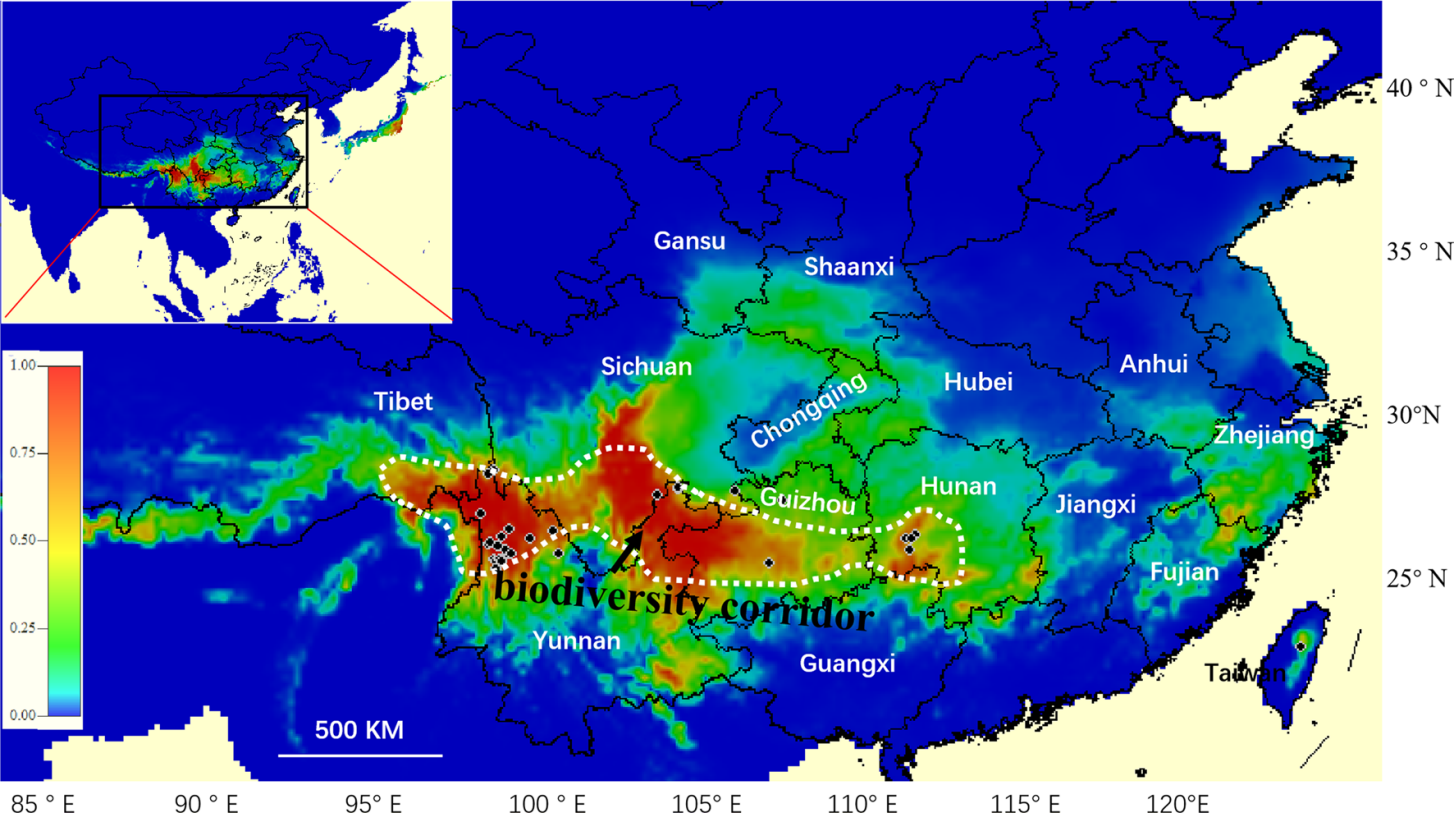
The prediction of the suitable distribution area of eight species within *O.sinensis*-species complex. *Note* Species occurrence were marked with black dots of the eight species

A prediction of the area suitable for eight species in the *O. sinensis-* species complex was obtained with the species distribution modeling method. Major suitable distribution areas (highlight with red colour) appear in southern and southeastern edge of the Hengduan Mountains, the Yunnan-Guizhou Plateau and local areas of the Xuefeng Mountains, and some suitable areas exist in eastern Taiwan and Fujian Province (Fig. 7). The main geographical distribution, especially in the southwest of China, predominantly present not sporadic but continuous large regions.

A biodiversity corridor hypothesis (生态长廊假说) is deduced for the *O. sinensis*-species complex based on their potential suitable distribution prediction. The *O. sinensis*-species complex evidently could have an entirely suitable distribution area from west to east, which could provide an excellent ecological environment for the spread and evolution of this unique group, so that it could form a rich diversity and radiation adaptation characteristics. This ecological corridor mainly starts from the Qinghai-Tibet Plateau in the west and extends to the Xuefeng Mountains in the east, passing through the Hengduan Mountains and the Yunnan-Guizhou plateau (Fig. 7).

## DISCUSSION

Both morphological observations and phylogenetic analyses support the distinctiveness of *Ophiocordyceps megala*. Our species, *O. megala* is similar in both sexual and asexual morphology to *O. macroacicularis* and *O. xuefengensis*. However, *O. macroacicularis* has smaller conidiogenous cells, with 30.4–42.0 µm long, and its conidia being shorter, 3.0 − 5.0 × 5— 8.0 µm long. *O. xuefengensis* and *O. megala* are more similar in stromata, host type, and habitat ecology, but *O. xuefengensis* has a dark greyish-brown colony, its conidia are citriform and Lotus-like in shape, and are frequently aggregated, while *O. megala* has a more single, less aggregated conidia. In addition, the stromata of *O. megala* are mostly smooth and brown, while those of *O. xuefengensis* have yellow microvilli.

Furthermore, *O. megala* is delimited by its long, large, and lignified stroma, and has a huge *Endoclita* host, with longer phialides and larger conidia, which distinguish it from the other species. Both morphological and phylogenetic analyses (5-locus and ITS sequence data, respectively) show that *O. megala* is a new species with a *Hirsutella*-like asexual morph. The discovery of *O. megala* has further enriched the species diversity in the *O. sinensis*-species complex.

We also suggest the common local name “ChongCaoWang” (虫草王) in Lanping County as its formal Chinese name; “Chongcaowang” expresses the huge morphological features.

The potentially suitable distributional regions are predicted to extend from the southeastern QTP to the Xuefeng Mountains with non-sporadically fragmented regions. Just as the Hengduan Mountain are hypothesized to be a corridor between the Palaearctic and Oriental regions, bridging the faunas of the north and south (Wu et al. 2017), we also propose that the Hengduan Mountains and Yunnan-Guizhou plateau are the biodiversity corridor for the *O. sinensis-* species complex.

## CONCLUSIONS

In our study, the phylogeny, species diversity and potential suitable distribution are systematically illustrated and discussed of the *O. sinensis*-species complex lineage. And we described *Ophiocordyceps megala* new to this lineage. The biodiversity corridor hypothesis is proposed based on the suitable distributions prediction of *O. sinensis*-species complex. And the high confidence predictions should have positive guiding significance for subsequent resource discovery. The detailed description and comparison of these 10 species also have a positive implication for the adaptive evolution of this important valuable group. As the limited information from the morphology and phylogeny, multi-omics research is very necessary for the variation and adaptation around the *O. sinensis*-species complex.

### Supplementary Information

**Additional file 1:** Table 1 Primer pairs for gene amplification and sequencing used in this study. Table 2 Description of Bioclim variables used in the suitable distribution prediction.

**Additional file 2:** Figure 1 The specimens of *Ophiocordyceps megala* from Myanmar.

## Data Availability

All specimens were deposited in the Yunnan Herbal Herbarium (YHH), all isolations were deposited in the Yunnan Fungal Culture Collection (YFCC). All sequences were submitted to GenBank and NMDC.

## Abbreviations

BPP: Bayesian inference posterior probability
*cox1*: The mitochondrial cytochrome coxidase subunit I
ITS: Internal transcribed spacer
QTP: The Qinghai–Tibetan Plateau
MLBS: Maximium likelihood bootstrap support.
nrSSU: Small subunit ribosomal RNA
nrLSU: Large subunit ribosomal RNA
PDA: Potato dextrose agar
*rpb1*: The largest subunit of RNA polymerase II
*rpb2*: The second largest subunit of RNA polymerase II
*tef*: Transcription elongation factor-1 alpha
YFCC: The Yunnan fungal culture collection
YHH: Yunnan herbal herbarium
