# Supplementary figures and images for: Molecular phylogenetics of the Ophiocordyceps sinensis-species complex lineage (Ascomycota, Hypocreales), with the discovery of new species and predictions of species distribution
Source: IMA Fungus. 2024 Feb 10;15:2. doi: 10.1186/s43008-023-00131-8 (PMC10858606; doi:10.1186/s43008-023-00131-8)

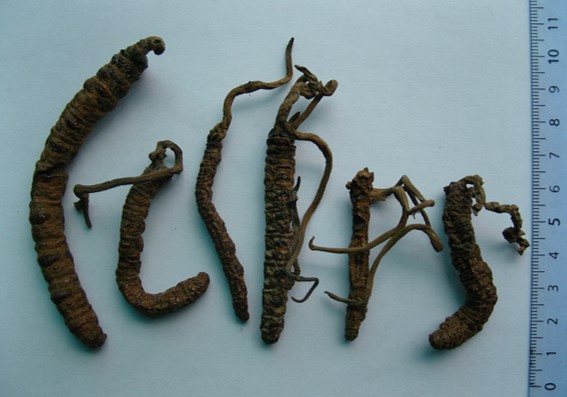

Supplement: Supplementary file 2 — Additional file 2: Figure 1 The specimens of Ophiocordyceps megala from Myanmar. [file 43008_2023_131_MOESM2_ESM.jpg]
